# Perspective of placenta derived mesenchymal stem cells in acute liver failure

**DOI:** 10.1186/s13578-020-00433-z

**Published:** 2020-05-24

**Authors:** Mahshid Saleh, Mohammad Taher, Amir Ali Sohrabpour, Amir Abbas Vaezi, Mohsen Nasiri Toosi, Maria Kavianpour, Zeinab Ghazvinian, Shahrokh Abdolahi, Javad Verdi

**Affiliations:** 1 Department of Tissue Engineering and Applied Cell Sciences, School of Advanced Technologies in Medicine, Tehran University of Medical Sciences, Tehran, Iran; 2 Gastroenterology and Hepatology, Tehran University of Medical Sciences, Imam Hospital Complex, Tehran, Iran; 3 Gastroenterology and Hepatology, School of Medicine Shariati Hospital, Tehran University of Medical Science, Tehran, Iran; 4 Department of Internal Medicine, School of Medicine, Alborz University of Medical Sciences, Karaj, Iran; 5 Internal Medicine, School of Medicine Liver Transplantation Research Center Imam, Khomeini Hospital Tehran University of Medical Sciences, Tehran, Iran

**Keywords:** Acute liver failure, Mesenchymal stem cells, Placenta, Cell therapy

## Abstract

Acute Liver failure (ALF) is a life-threatening disease and is determined by coagulopathy (with INR ≥ 1.5) and hepatic encephalopathy as a result of severe liver injury in patients without preexisting liver disease. Since there are problems with liver transplantation including lack of donors, use of immunosuppressive drugs, and high costs of this process, new therapeutic approaches alongside current treatments are needed. The placenta is a tissue that is normally discarded after childbirth. On the other hand, human placenta is a rich source of mesenchymal stem cells (MSCs), which is easily available, without moral problems, and its derived cells are less affected by age and environmental factors. Therefore, placenta-derived mesenchymal stem cells (PD-MSCs) can be considered as an allogeneic source for liver disease. Considering the studies on MSCs and their effects on various diseases, it can be stated that MSCs are among the most important agents to be used for novel future therapies of liver diseases. In this paper, we will investigate the effects of mesenchymal stem cells through migration and immigration to the site of injury, cell-to-cell contact, immunomodulatory effects, and secretory factors in ALF.

## Introduction

Liver is one of the largest vital organs in human body that controls various biological processes, including the production of multiple hormones, storage of glycogen, neutralization of toxins and drugs, control of metabolism, metabolism of urea, and synthesis of plasma protein. Typically, most physiological features of liver function are controlled by liver cells or hepatocytes; therefore, the loss of hepatocytes is the main cause of liver failure. Several diseases related to malfunction of the liver are caused by damage to or loss of hepatocytes, including viral hepatitis, fatty liver disease, drug and toxin-induced liver injury, hepatocellular carcinoma, and hepatic abnormalities associated with autoimmunity and cirrhosis [1]. In adults, the liver weighs nearly 1.4 kg (3.1 lb) and lies to the right of the abdomen below the diaphragm [2]. Each year, many people worldwide develop liver disease. Acute liver injury (ALI), acute liver failure (ALF), acute on chronic liver failure (CLF), and inherited metabolic liver diseases are examples of liver diseases [3].

## Liver failure

Liver failure is a clinical syndrome diagnosed with clinical signs of jaundice, ascites, hepatic encephalopathy and a tendency for bleeding due to liver damage. This syndrome can occur for a variety of reasons, including viral hepatitis, autoimmune hepatitis and liver damage [4]. Approximately 1.6 cases per million people worldwide develop this serious disease annually, which in turn results in high costs and mortality [5, 6]. Patients with drug induced liver injury are associated with some degree of ascites, encephalopathy, coagulopathy of any grade (PT (prothrombin time), INR (international normalized ratio)) as well as impaired liver function (AST (aspartate aminotransferase), ALT(alanine transaminase), TBIL (Total bilirubin Indirect level), ALB (Albumin)). Liver failure is divided into three forms as follows. ALF within 48 h to several days with jaundice, coagulopathy and encephalopathy; acute-on-chronic liver failure (ACLF) with a background of chronic liver disease leading to rapid progression of liver injury and associated with jaundice and ascites; and CLF occurring within months to years [7].

## Acute liver failure (ALF)

ALF is an unpredictable and potentially catastrophic condition often encountered in intensive care units, with more than 2500 cases reported each year in the United States. The progression potential of acute hepatic dysfunction toward multi-organ failure demands rapid diagnosis and management of the disease. Due to a set of hepatic and non-hepatic complications, ALF indirectly leads to immediate follow-up for liver transplantation [8]. ALF, formerly known as fulminant hepatic failure, means the development of hepatocellular disorders such as coagulopathy and encephalopathy with INR ≥ 1.5 in patients without a history of liver disease within 26 weeks. More than half of the cases of ALF progression require liver transplantation and significant improvements have been reported in the last decade after liver transplantation. ALF mortality is usually due to intracranial hypertension (ICH) and infection [9–11]). However, patients with varying degrees of hemodynamic disorders and renal failure have also been reported [12, 13]. Clinically, the patients show coagulopathy, jaundice and hepatic encephalopathy. The period between the onset of the first clinical symptoms and hepatic encephalopathy is crucial in determining the prognosis of these patients [14, 15]. There are obvious differences in the development mechanisms of early ALF. The three main factors determining the prognosis of this disease include metabolic problems leading to the loss of liver cells, secretion of toxins and mediators from the liver tissue, and capacity of the remaining hepatocytes to repair the liver [15, 16].

Common treatments are therapies that are often meant to improve the complications of acute liver failure (ALF). Multiple organ failure (MOF) and severe infection are the most prevalent factors of mortality in these patients. Therefore, management of treatment for ALF patients should focus on the handling and prevention of infection [17]. ALF patients with severe hepatic encephalopathy, those with renal failure and patients who have any of SIRS criteria use broad-spectrum antibiotics [18]. Application of vasoconstrictors and dialysis reduce the incidence of cerebral edema [19]. In case of hepatic encephalopathy, the patient is transferred to ICU and ventilator devices are used to regulate the level of blood gases patients with ALF have qualitative and quantitative coagulation abnormalities. In control of bleeding and during invasive procedures, there is indication for FFP and platelet administration [20]. To prevent gastrointestinal bleeding in ALF, patients admitted to ICU are treated with H2 blockers or proton pump inhibitors (PPI) [21]. Patients with ALF are at risk of hypovolemia for a number of reasons, including poor oral fluid intake, vomiting, and vasodilation, in which case bolus fluids are used and level of fluids is frequently maintained if necessary to keep serum sodium levels and prevent fluid overload [22]. In addition to the mentioned treatments, 10–20% glucose is administered when glycemic target is 140 mg/dL and Na level is 135–145 mmol/L, as well as N-acetylcysteine and stress ulcer prophylaxis agents [17].

## Etiology

A wide variety of factors cause ALF (Table 1) [23–25]. The most common causes of this disease are viral infections and drug-induced liver inflammation. In Asia and parts of Europe, mainly viral hepatitis agents are involved and acetaminophen is the predominant factor in countries such as USA and Australia [26].

**Table 1:** Etiology of ALF

Other etiologies of ALF	The most common global agents of ALF
Hypoxia-induced liver injury Acute Budd-Chiari Syndrome Veno-occlusive Disease Wilson’s disease Mushroom Ingestion Sepsis Autoimmune hepatitis Acute fatty liver of pregnancy HELLP (hemolysis, elevated liver enzymes, low platelet) syndrome Heat stroke Malignant infiltration of the liver	Viral hepatitis Drug-induced hepatitis

## Immune system in patients with ALF

Impaired function of both humoral and innate immunity is implicated in the pathophysiology of ALF [27].

The mechanism of ALF begins with necrosis of hepatocytes [28]. Oxidative stress is triggered when liver injury is caused by factors such as viral infections, alcohol consumption, drug intoxication, autoimmune diseases, herbal remedies and many other factors [29–31]. Oxidative stress results in the production of reactive oxygen species, which in turn activates the Janus kinase (JNK) signaling pathway [32] and generates damage-associated molecular patterns (DAMPs), followed by liver inflammation. Liver inflammation is a major factor in immunopathology of several hepatic diseases [33, 34]. DAMPs activate hepatic macrophages (Kupffer cells (KCs)) and induce the formation of inflammasome [32, 35] that eventually leads to the secretion of IL-1, IL-18, and caspase 1 [32]. DAMPs are detected by Kupffer cells [33, 36] that express a large number of DAMP receptors, including TLR4, TLR9, and RAGE [36]. KCs are activated in this process and release inflammatory cytokines such as TNFα, oxygen radicals, and chemokines such as CCL2 under the effect of inflammatory signals. The presence of inflammatory factors mobilizes inflammatory cells such as neutrophils and monocytes and thereby increases inflammation [33, 34].

## Hepatic encephalopathy in ALF

Hepatic encephalopathy (HE) is a function of neurotoxins that reach the brain through the bloodstream [37]. Various factors such as blood ammonia levels, infection, necrotic liver, toxins, and systemic inflammatory response syndrome (SIRS) can lead to HE [38]. In normal conditions, the ammonia produced in the body is efficiently excreted by the liver through the urea cycle and glutamine synthesis and thus a small amount of ammonia remains in hepatic vessels. In ALF, ammonia levels rise in the hepatic vein, and the liver loses the ability to release ammonia from the hepatic veins. The muscles and brain begin ammonia detoxification through glutamine synthesis. Therefore, both of these tissues are considered as an ammonia scavenging and glutamine releasing organs [39].

Tissue damage is the first factor triggering SIRS reaction. As explained above, the injury leads to the release of inflammatory mediators such as DAMPS, TNFα, IL-6, and IL-18. Inflammatory cells such as lymphocytes and monocytes reach the damage site and enhance the inflammatory response. Coagulation factors as well as primary and secondary homeostasis also become involved and result in SIRS reaction [38]. These reactions are associated with the development of HE [40, 41], bacteremia [42] and, in some cases, infection [41, 43].

Compensatory anti-inflammatory response syndrome (CARS) occurs in reaction to SIRS, leading to the secretion of anti-inflammatory factors (including IL-10 and SPLI) from hepatic macrophages during the early stages. This reaction is meant to alleviate the inflammatory status [44, 45]. Both of these reactions eventually lead to dysregulation of the immune system and defective immune responses to microbial agents [46, 47].

## Mesenchymal stem cells and their secreted factors

MSCs are fusiform non-hematopoietic cells capable of adhering to plastic surfaces, which can be isolated from various tissues, including placenta, umbilical cord, bone marrow, adipose, and other tissues [48].

Despite their morphological and phenotypical similarities, MSCs have different regeneration potentials [49], which is due to the microenvironment and cellular niches affecting their fate [50]. The number of stem cells in many adult tissues is small and isolation of them is associated with several risks; for example, the cells exhibit a limited capacity for differentiation and proliferation after removal from the body, making it difficult to produce large numbers of stem cells [51]. In comparison to adipose tissue and BM, in which MSCs are affected by donor’s age, placenta is a rich source of stem cells [52] and high differentiation capacity and pluripotentiality of placental cells are related to their origin [53]. PD-MSCs have a higher proliferative potential than BM-MSCs [54] which reduces the number of passages to reach a large number of cells as well as the risk of cell aging [55, 56]. Among MCSs, PD-MSCs have a higher potential for in vitro proliferation and differentiation of hepatocytes [57]. Human BM-MSC cells are involved in neovasculogenesis and synergize with endothelial colony forming cells (ECFCs) to create microvessels in vivo [58, 59]. BM-MSC cells serve as the gold standard for bone and cartilage repair [60]. Adipose tissue-derived mesenchymal stem cells (AD-MSCs) are isolated from adipose tissue by liposuction, are capable of differentiation to hepatocyte-like cells in the presence of HGF, FGF-1, and FGF-4 factors and participate in the regeneration of hepatocytes and vasculogenesis [61]. Wharton’s jelly mesenchymal stem cells (WJ-MSCs) exhibit stemness and pluripotential properties and have been shown to generate various types of neurons and connective tissue cells [62, 63].

Umbilical cord-derived mesenchymal stem cells (UC-MSCs) have been recognized as low-immunogenicity cells because of their immunomodulatory properties. UC-MSCs are involved in neovascularization and differentiation into hepatocyte-like cells [64, 65]. Umbilical cord blood has always been considered as a source of hematopoietic stem cells (HSCs) [66]. The phenotypic characteristics of UC-MSCs are consistent with BM-MSC cells [67].

Dental tissue‐derived mesenchymal stem cells (DP-MSC) have limited differentiation capacity relative to BM-MSCs [68]. Dental pulp stem cells (DPSCs) are dental stem and progenitor cells that are capable of self-renewal and differentiation, which differentiate into neurons and adipocytes in addition to odontogenic cells [69, 70].

The definition of MSCs according to International Society for Cell Therapy (ISCT) is as follows: MSCs are (1) able to bind plastic surfaces, (2) able to differentiate into all three classes of chondrocytes, adipocytes and osteocytes in vitro, and (3) capable of expressing CD73, CD90, and CD105 markers but not hematopoietic markers like CD45, CD14, CD19, CD34, and HLA-DR [71]. MSCs release numerous factors such as vascular endothelial growth factor (VEGF), insulin-like growth factor 1 (IGF-1), basic fibroblast growth factor (bFGF), nerve growth factor (NGF), Transforming growth factor beta-1 (TGF-b1), placental growth factor (PGF), stromal cell-derived factor 1 (SDF-1/CXCL12), monocyte chemoattractant protein-1 (MCP 1/CCL2), hepatocyte growth factor (HGF), interleukin-6 (IL-6), IL-8, IL-10, IL-13, G-CSF and GM-CSF [72–75].

There are various tissue-specific factors in MSCs depending on the tissues from which MSCs are isolated. For example, factors such as HGF, bFGF, and IL-6 are mainly secreted by MSCs isolated from placental tissue or interferon-γ (IFN-γ), tumor necrosis factor α (TNF-α), interleukin-1 alpha (IL-1α), and interleukin-1 beta (IL-1β) secreted by MSCs from Menstrual blood-derived stem cells (MenSCs) [76, 77]. Hence, it can be said that the selection of mesenchymal stem cells extracted from tissues is an important consideration in the treatment of diseases with respect to the secretory factors they produce.

## Placenta-derived mesenchymal stem cells

Embryonic stem cells are isolated from embryonic tissues, especially multiple extraembryonic tissues. Tissues such as amniotic fluid, Wharton’s jelly, amnion, chorion, embryonic membrane and placenta have MSCs. The placenta is one of the largest organs with an essential role in the development of the fetus, which plays a role in the secretion of nutrients for the fetus and immune protection (tolerance) of it. It has recently been observed that PD-MSC are a new alternative source of MSCs for regenerative therapies [78]. Studies have shown that PD-MSCs possess self-renewal capacity, have multilineage differentiation, lack ethical problems, are accessible, abundant, and show strong immunosuppressive effects [79–81]). In addition, placental tissue derived from the fetus is voluminous and can be easily manipulated to increase the number of MSCs, which exceeds the number of MSCs present in bone marrow and adipose tissue [81, 82]. Another advantage of these placental stem cells is that we do not require an invasive method to isolate them, whereas invasive methods are needed to isolate adult MSCs [78].

Typically, PD-MSCs can maintain a high proliferative capacity in culture medium for at least 20 passages [83]. Some studies have recently suggested the differentiation of PD-MSCs into hepatocyte-like endodermal cells [57, 84]. Investigations have shown that many perinatal resources of MSCs such as amniotic membrane (AM), chorionic plate (CP), parietal decidua [85], and umbilical cord (UC) have advantages relative to adult sources, including bone marrow (BM) [86–88]. The MSCs isolated from these tissues have their own characteristics as follows. VCAM1 is a biomarker of chorionic plate with unique immunosuppressive activity that plays an important role in immune responses [86]. CP-derived mesenchymal cells copiously secrete HGF and VCAM1. Parietal decidua derived mesenchymal stem cells (DMSCs) [85] show a high secretion of Ang1 and VEGF but the lowest secretion of TGFβ1. Umbilical cord (UC) derived MSCs have a high secretion level of IGF1 and amniotic membrane (AM) derived MSCs highly release PEG2 and TGFβ1 [89]. Considering the above statements, we show in this research that amniotic membrane-derived mesenchymal stem cells may be effective in treatment of premature ovarian aging due to overexpression of PEG2 and TGFβ1, CP-derived MSCs could be used for angiogenic therapy because of pro-angiogenic activity, and parietal decidua derived MSCS [85] might be useful for the treatment of vital organ ischemia, and UC-MSCs may be used for other therapies because of secreting a large number of factors [90].

## Possible disadvantages of MSCs

Most animal and human studies on MSCs have indicated therapeutic effects of these cells. However, there is evidence for low engraftment of MSCs due to short-term viability after injection [77, 91]. MSCs are trapped in the lung after injection and a lower number of these cells may reach their destination [92]. Therefore, the reduction of cell loss during migration is an advantage of topical over intravenous injection [93]. Several studies have indicated that a single injection of MSCs is safe for the patient and does not stimulate the immune system, but re-injection of MSCs may lead to the generation of alloantibodies [94]. In addition, the FBS that is used to grow MSCs could induce an immune response in the patient [95]. In general, MSCs show a dual behavior when faced with tumors and PD-MSCs are no exception in this regard. For example, some in vitro studies have indicated that UC-MSCs increase the expression of proliferating cell nuclear antigen (PCNA) [96], induce the proliferation promoting genes like EPGN/MZT2A, downregulate transcription factors associated with the suppression of tumor development such as TAL1/FOS/EGR1/KLF10, which stimulates different tumor populations [97]. Pursuant to this dual role of PD-MSCs, one study introduces the antitumor role of these cells in a particular type of tumor but suggests a promoter role in another type. WJ-MSCs have an antitumor role in the face of squamous cell carcinoma in vitro, but stimulate the growth of cancer in vivo [98].

## Therapeutic approaches for acute liver failure & PD-MSCs advantages

Clinicians have observed that a number of patients with ALF may recover spontaneously and that the clinical outcome of these patients largely depends on the balance between loss and repair of hepatocytes [99]. The damaged hepatocytes are rapidly replaced by normal hepatocytes in moderate disease, but in case of severe injury and widespread death of hepatocytes, the repair capacity of remaining hepatocytes may not be complete and lead to the deployment of liver progenitor cells (LPC) that act as hepatocytes [100]. In most ALF patients, these progenitor cells are insufficient to repair and replace hepatocytes, eventually leading to the adoption of limited therapeutic approaches by physicians [101]. Today, liver transplantation is the only way to treat liver failure patients. However, liver transplantation has failed for a number of reasons such as lack of proper organs, high costs, and the administration of immunosuppressive agents for long periods of time. Other treatment strategies include bioartificial liver with less hepatocytes and drug therapy [102]. Hepatic failure is a disastrous consequence of liver loss, in which the repair of residual hepatocytes is not performed in a timely and appropriate manner, resulting in increased mortality [103]. Massive hepatic necrosis in acute liver failure [97] is caused by sudden loss of hepatocytes due to a variety of acute injuries induced by hepatotoxic drugs, immune system attack, and viral infections [104–106]. While most hepatocytes are completely destroyed in ALF, the circulating Bone Marrow-derived cells and endogenous hepatocyte progenitor cells can rapidly regenerate the liver [107]. Cell-based therapies have been promising in regenerative medicine. MSCs can be important sources of alternative therapy because of various properties such as self-renewal, proliferation and differentiation [108].

## Mechanisms of PD-MSC effect on acute liver injury

**Fig. 1:**
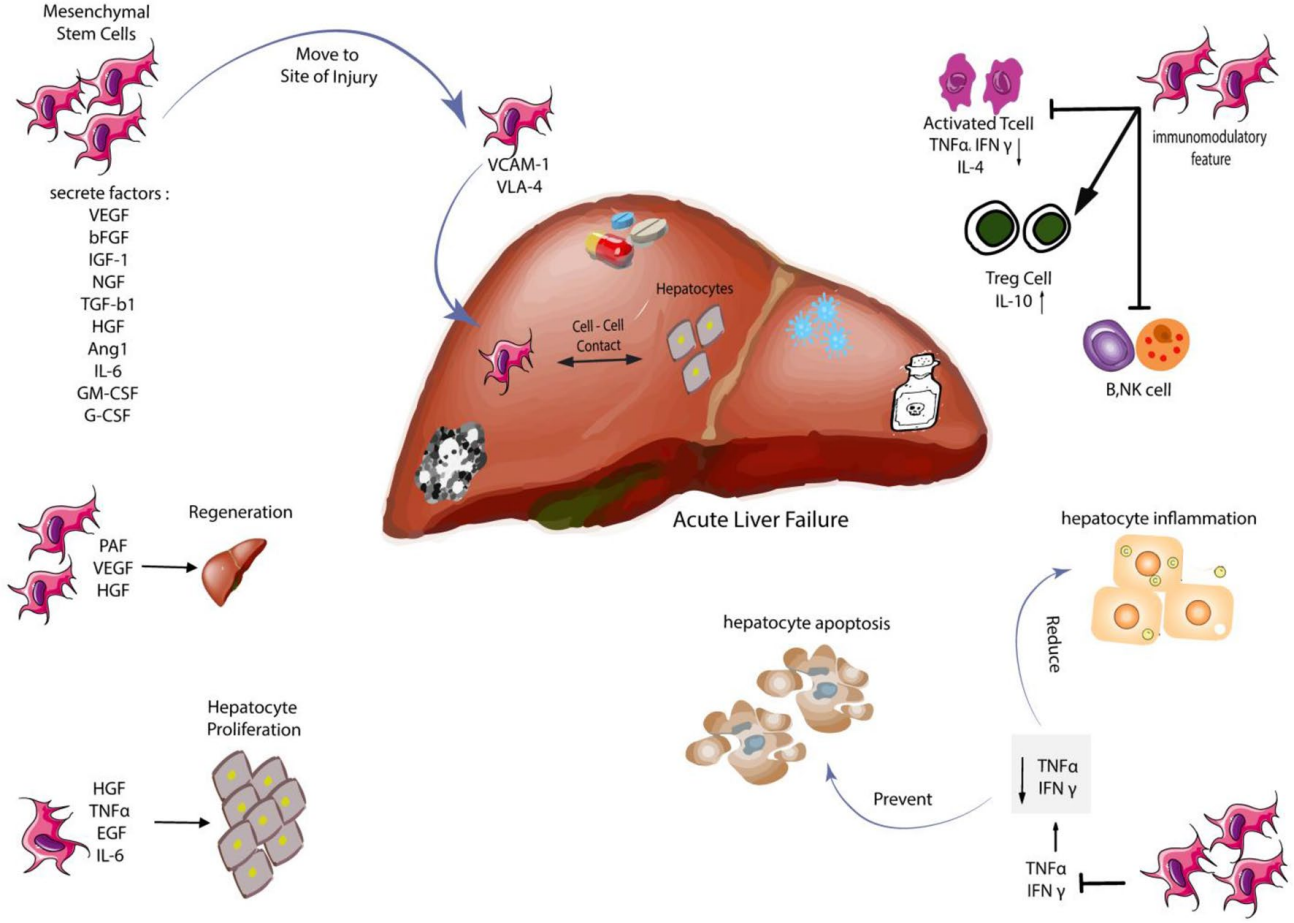
Mesenchymal stem cells and its effects on acute liver failure

The precise mechanism of MSCs in ALF is not completely understood [109]. According to several studies, it can be stated that placenta-derived mesenchymal stem cells (PD-MSC) are able to affect the liver damages in several ways:

1. PD-MSCs are recruited to the damaged area by VCAM-1 and VLA-4 adhesion molecules [104, 110, 111] affecting the remaining hepatocytes through cell–cell contact and secretion of TGF-α, EGF, HGF, and VEGF tropic factors [112, 113].
2. PD-MSCs have immunomodulatory properties and increase Treg cells, modulating the immune system as well as suppressing activated T-cells, NK cells, B-cells and IL-10 production [113, 114].
3. PD-MSCs decrease the inflammation of hepatocytes and prevent their apoptosis by suppressing TNFα and IFNγ, which leads to the regeneration of hepatocytes by releasing HGF, IL-6, PAF and VEGF [115, 116].
4. MSCs are capable of secreting various angiogenic factors, including VEGF, SDF-1α, and MMP1, which promote angiogenesis [117–119].
5. In addition to their immunomodulatory properties, MCSs differentiate into vascular cells and pericytes in vivo [117]. They also have the potential to differentiate into hepatocyte-like cells both in vivo and in vitro, leading to improvement of liver damage (Fig. 1) [115, 120].

A majority of studies have used the intravenous route to inject MSCs, after which most MSCs are trapped in lungs in the early stages [121, 122]. After 24 h, MSCs move toward other organs (especially the liver and spleen) and settle in them [123]. They also migrate to damaged tissues [123]. For instance, in a study on patients with cirrhosis, MSCs labeled with ^111^IN-Oxine were detected in the liver after 48 h (through radioactivity assay) where they remained for 10 days [124]. Elimination of MSCs may be related to the immune system, which does not rule out the functional effect of these cells. One study has reported that phagocytosis of dead MSCs induces the production of regulatory macrophages modulating the immune response by producing IL-10 factors [125, 126]. Moreover, a small fraction of these cells that have been spared elimination could be responsible for the therapeutic effects [126].

MSCs play a critical role in liver regeneration because of their ability to produce and regulate platelet-activating factor (PAF), hepatocyte growth factor (HGF) and vascular endothelial growth factor (VEGF) [104]. Several studies have demonstrated the significance of MSCs in liver diseases. MSCs have been used in various investigations on ALF in both animal models [127, 128] and clinical trials [129, 130]. Nevertheless, the precise mechanism of the function of these cells remains unclear. Since MSCs are able to move to the site of injury and inflammation [131] as well as being capable of proliferating and differentiating into hepatocytes [132, 133], they play an essential role in regenerative therapies. MSCs show immunomodulatory feature because they do not express stimulatory molecules or HLA II [134] and are therefore a good source for allogeneic and autologous transplantation.

Several studies have shown that MSCs secrete tropic factors and can be effective in reducing inflammation, fibrosis and apoptosis of liver cells as well as repairing damaged tissue by stimulating angiogenesis [74].

High migration ability is a major advantage of PD-MSCs. Migration involves the movement of MSCs toward damaged and inflamed sites through interactions between MSCs with cytokines and adhesion molecules secreted from the injured tissue environment [135]. Migration of MSCs has been investigated in both animal [136] and in human studies [137]. For example, various researches have revealed that MSCs express adhesion molecules and integrins such as VCAM-1 and VLA-4, which are composed of CD29 and CD49d components. Compared with BM-derived mesenchymal stem cells (BM-MSCs), placental MSCs express a higher level of VLA-4 and animal studies have indicated MSCs binding to endothelial cell surface markers such as P-selectin and VCAM-1, which is indicative of the high implantation capacity of PD-MSCs into damaged tissue [111, 138]. A clinical trial of cirrhotic patients showed that ^111^In-oxine-labeled MSCs were trapped in the lungs in the early hours after injection through peripheral blood and that they left there after 48 h and migrated to the liver and spleen, remaining in these tissues for 10 days [137].

There are various mechanisms in the creation of an immunologically safe environment by placenta for the fetus [139]. This feature is a strong advantage for PD-MSC cell therapy in allogeneic transplantation, which prevents graft rejection, stabilizes the transplant and drives MSCs, including BM-MSCs and amniotic fluid-derived MSCs (AF-MSCS), toward the site of injury [140]. Embryonic-derived MSCs are also capable of migrating to the placenta and blood brain barrier (BBB) [141]. It can be argued that the beneficial effects of MSCs in liver diseases (including ALF) are not limited to hepatocyte repair, but rather the tropical factors released by them modulate the deleterious effects of the immune response [142]. The immunosuppressive effects of MSCs on the secretion of TNFα and IFN γ prevent from apoptosis of hepatocyte cells and reduce hepatic inflammation, and the suppression of these cytokines appears to be systemic [143]. MSCS in mice with ALF suppress activated T-cells, decreasing the inflammatory cytokines TNFα, γ IFN and IL-4 and exerting their immunosuppressive effects by increasing IL-10 levels [143, 144]. Cells such as natural killer T (NKT) are of high importance in the pathogenesis of ALF and are immunomodulatory targets mediated by MSCs along with dendritic cells (DCs), macrophages and T-cells [143, 145].

HGF is one of the most important factors in the repair of hepatic tissue, which is secreted by MSCs. Hepatocyte growth factor is an effective mitogen for hepatic tissue repair that is dependent on c-met receptor during tissue damage [146]. The HGF/c-met signaling pathway is essential for liver repair and implantation of MSCs in the affected area [147]. Many studies have reported the protective effects of HGF/c-met signaling pathway on liver injury [148, 149]. HGF as well as other factors like TNFα and EGF is considered a mitogenic factor associated with hepatocyte proliferation [112, 150]. On the other hand, HGF together with NGF factor secreted by MSCs induces apoptosis of Hepatocyte Stellate Cells (HSC), indicating the antifibrotic property of these cells [151–153]. Many studies have shown that angiogenesis plays a crucial role in hepatic repair so that the injection of anti-angiogenic factors such as anti-VEGF inhibits hepatic repair [154, 155] but factors such as bFGF enhance it [156]. VEGF boosts angiogenesis and contributes to the healing process [157]. Angiogenesis is essential for wound healing, regeneration and organogenesis [158]. IL-6 binds to gp80 and gp130 receptors, which activate the JAK pathway and in turn phosphorylate tyrosines in the intracellular domain of gp130, subsequently activating the MAPK pathway and STAT 1 and 3 transcription factors that lead to hepatocyte proliferation [159–161]. Recent experiments on animal models have shown that IL-6 and TNF-2 are involved in regeneration of liver mass [162].

## Conclusion

Limited information is available on the repair mechanism of MSCs in various diseases; therefore, further in vivo studies provide a broad perspective for MSCs use in clinical practice. Choosing the right cell, determining the proper dose, selecting the appropriate injection site and timely injection can help improve the function and implantation of MSCs in the target tissue, and they can be highly important and applicable for further research in the future. In this review paper, we concluded that PD-MSCs can be considered as a good allogeneic source for ALF in future because of their safety, easy accessibility, lack of immune system stimulation, secretion of appropriate factors for liver tissue and healing properties.

## Data Availability

Not applicable.

## Abbreviations

MSCs: Mesenchymal stem cells
PD-MSCs: Placenta-derived mesenchymal stem cells
ALF: Acute liver failure
ACLF: Acute-on-chronic liver failure
CLF: Choronic liver failure
ICH: Intracranial hypertension
DAMPs: Damage-associated molecular patterns
SIRS: Systemic inflammatory response syndrome
CARS: Compensatory anti-inflammatory response syndrome
ISCT: International Society for Cell Therapy
VEGF: Vascular endothelial growth factor
IGF-1: Insulin-like growth factor 1
bFGF: Basic fibroblast growth factor
NGF: Nerve growth factor
TGF-b1: Transforming growth beta-1
IFN-γ: Interferon-γ
TNF-α: Tumor necrosis factor α
IL-1α: Interleukin-1 alpha
IL-1β: Interleukin-1 beta
PGF: Placental growth factor
SDF-1/CXCL12: Stromal cell-derived factor 1
MCP 1/CCL2: Monocyte chemoattractant protein-1
HGF: Hepatocyte growth factor
G-CSF: Granulocyte-colony stimulating factor
GM-CSF: Granulocyte-macrophage colony stimulating factor
AM: Amniotic membrane
CP: Chorionic plate
UC: Umbilical cord
LPC: Liver progenitor cells
PAF: Platelet-activating factor
VLA-4: Very late antigen 4
BBB: Blood brain barrier
HSC: Hepatocyte stellate cells
JAK: Janus kinase
